# BNT162b2 vaccination reduced infections and transmission in a COVID‐19 outbreak in a nursing home in Germany, 2021

**DOI:** 10.1111/irv.13051

**Published:** 2022-09-09

**Authors:** Emily Dorothee Meyer, Mirco Sandfort, Jennifer Bender, Dorothea Matysiak‐Klose, Achim Dörre, Gerhard Bojara, Konrad Beyrer, Wiebke Hellenbrand

**Affiliations:** ^1^ Department of Infectious Disease Epidemiology, Postgraduate Training for Applied Epidemiology (PAE) Robert Koch Institute Berlin Germany; ^2^ European Programme for Intervention Epidemiology Training (EPIET) European Centre for Disease Prevention and Control (ECDC) Stockholm; ^3^ Department of Infectious Diseases, Nosocomial Pathogens and Antibiotic Resistances Unit Robert Koch Institute Berlin Germany; ^4^ European Programme for Public Health Microbiology Training (EUPHEM) European Centre for Disease Prevention and Control (ECDC) Stockholm Sweden; ^5^ Department of Infectious Disease Epidemiology, Immunization Unit Robert Koch Institute Berlin Germany; ^6^ Local Public Health Authority Osnabrück Osnabrück Germany; ^7^ Department of Infectious Diseases Public Health Agency of Lower Saxony Hanover Germany

**Keywords:** COVID‐19 vaccines, disease outbreaks, nursing homes, SARS‐CoV‐2, vaccine effectiveness

## Abstract

**Background:**

A SARS‐CoV‐2 outbreak was detected in a nursing home in February 2021 after residents and staff had received two doses of BNT162b2 vaccine in January 2021.

**Methods:**

Nursing home staff, long‐term residents and day‐care receivers were included in a retrospective cohort study. We calculated attack rates (AR), secondary AR (SAR) and their 95% binomial confidence interval (CI), and we compared them using Fisher's exact test or chi‐squared test, depending on the sample size. We used Poisson regression with robust error estimates to calculate vaccine effectiveness against SARS‐COV‐2 infections. We selected variables based on directed acyclic graphs. As a proxy for viral load at diagnosis, we compared the mean Ct values at diagnosis using t tests or Mann–Whitney U tests.

**Results:**

The adjusted vaccine effectiveness against infection was 56% (95% CI: 15–77%, p = 0.04). Ct values at diagnosis were higher when intervals after receiving the second vaccination were longer (>21 vs. ≤21 days: 4.48 cycles, p = 0.08). The SAR was 67% lower in households of vaccinated (2/9 [22.2%]) than of unvaccinated infected staff (12/18 [66.7%]; p = 0.046). Vaccination rates were lowest among staff with close physical contact to care‐receivers (46%). The highest AR in vaccinated staff had those working on wards (14%).

**Conclusions:**

Vaccination reduced the risk for SARS‐CoV‐2 infection, viral load and transmission; however, non‐pharmaceutical interventions remain essential to reduce transmission of SARS‐CoV‐2 infections, even for vaccinated individuals. Vaccination coverage of staff ought to increase reduction of infections among themselves, their household members and residents.

## 1 INTRODUCTION

While COVID‐19 case fatality was <0.1% in under the age of 50 years,^1^ it was 13% in outbreaks of SARS‐CoV‐2 in nursing homes in Germany between January 2020 and February 2021.^2^ Hence, nursing homes were prioritized for COVID‐19 vaccination, which began in Germany in December 2020.^3^ However, the vaccine effectiveness (VE) in such institutionalized setting, particularly in aged and comorbid care receivers, was unknown.

We report on a SARS‐CoV‐2 Alpha (B.1.1.7) outbreak among day‐care receivers and long‐term residents and staff of a nursing home in Osnabrück, Germany, January to March 2021. Due to vaccine prioritization policies, only residents of long‐term care wards and all staff of nursing homes were eligible for voluntary COVID‐19 vaccination.^4^ Hence, all day‐care receivers, some long‐term residents and some staff were unvaccinated during the outbreak.

This study describes the epidemiology of the outbreak and the undertaken control measures. We assess vaccination coverage (VC) and attack rates (AR) by occupational groups, VE against SARS‐CoV‐2 Alpha infections and vaccine effects on viral load and secondary transmission.

## 2 METHODS

### 2.1 Study population and definitions

We conducted a retrospective cohort study and included all care‐receivers and staff working in the nursing home between 03 January (symptom onset of first case) and 18 March 2021 (2 weeks after diagnosing the last case). We defined cases of the outbreak as care‐receivers or staff who had a positive SARS‐CoV‐2‐PCR result between 03 January 2021 and 18 March 2021. We defined the date of diagnosis as the earlier date of either symptom‐onset or sampling of the first positive test (PCR or rapid antigen detection tests [RADT]). We grouped staff according to their degree of contact with care‐receivers into staff with close physical contact (nursing specialist, nursing assistants, nursing trainees and interns), staff working on wards (chaplains, administrative care and cleaner) and staff working outside of wards (administration, kitchen and housekeeping).

To calculate secondary attack rates (SAR) in households of infected staff, we collected the number of household members and their PCR results from the local health authority database. Secondary cases were defined as SARS‐CoV‐2 PCR‐positive household members diagnosed 1–14 days after the diagnosis of the corresponding staff index case. All household members of infected staff were PCR‐tested on days 5 and 10 of their quarantine. We excluded household members with a PCR‐confirmed SARS‐CoV‐2 infection within 6 months prior or who quarantined separately from infected staff, of which none were diagnosed with a SARS‐CoV‐2 infection prior to this outbreak. One household outside of the administrative district was excluded because data was unavailable.

### 2.2 Descriptive and analytical epidemiology

We estimated AR of SARS‐CoV‐2 infections with their 95% confidence interval (CI) and compared AR between the vaccinated and unvaccinated cohort using chi‐squared or Fisher's exact tests, respectively.

We estimated VE as VE = 1 − RR, for which the relative risk (RR) for SARS‐CoV‐2 infection in vaccinated versus unvaccinated individuals was estimated via Poisson regression.^5^ We calculated 95% CI with robust standard errors. Our variable selection included vaccination status as the exposure and SARS‐CoV‐2 infection as the outcome. Based on directed acyclic graph modelling (Supporting Information S1), we further included the status (long‐term residents, day‐receivers or staff) and sex. We could not include the risk perception for an infection before vaccination because the respective data were unavailable.

### 2.3 Determination of viral load

We assessed viral load at diagnosis using the Ct value for the ORF1AB gene at the first positive PCR as a proxy. Depending on the data distribution, we used t tests or Mann–Whitney U tests to compare if the mean viral load differed between vaccinated versus unvaccinated, >14 days versus ≤14 days and >21 days versus ≤21 days between receiving the second vaccination and sampling.

### 2.4 Contact network analysis

Using the date of diagnosis with contact information collected by the local public health authority (LPHA), we inferred a possible contact network and presumable transmission chains which were visualized with the R package epicontacts.^6^

## 3 RESULTS

### 3.1 Study population

The nursing home comprised one ward for day‐care and seven for long‐term care with a total of 128 staff members, 100 long‐term care residents and 24 day‐care receivers. The median age was 49 years among staff (interquartile range [IQR] 32–58) and 87 years among care‐receivers (IQR 83–92). Of the care‐receivers and staff, 97/124 (77%) and 113/128 (88%), respectively, were female. Ninety‐five of 124 (77%) care‐receivers and 72/128 (56%) staff members were vaccinated with two doses BNT162b2 (Comirnaty, Pfizer/BioNTech) on 04 January and 25 January 2021 during vaccination events in the nursing home. Vaccination status did not correlate with sex (p = 0.90).

### 3.2 Outbreak description

PCR confirmation of the first SARS‐CoV‐2 infection in a long‐term resident initiated an outbreak investigation on 03 February 2021. Between 03 January 2021 and 18 March 2021, 50 SARS‐CoV‐2 cases were detected. Four long‐term residents (1/4 [25%] vaccinated) were hospitalized, and five died (2/5 [40%] vaccinated) from or with COVID‐19 (Table 1). The AR (8/24 = 33% vs. 26/100 = 26%, p = 0.46), hospitalization rate (3/8 = 38% vs. 1/26 = 4%, p = 0.03) and death rate (2/8 = 25% vs. 3/26 = 12%, p = 0.57) of cases were higher among day‐care receivers than long‐term residents.

**TABLE 1:** Outcomes of a SARS‐CoV‐2 Alpha outbreak in a nursing home in Osnabrück, Germany, Jan–Mar 2021 stratified by vaccination status among residents, staff, and all

	Vaccination				Case		Hospitalization		Death	
			n	Vaccination coverage	n	AR^a^	n	% of cases^b^	n	% of cases^b^
Care‐receivers	Day‐care	No	24	0%	8	33%	3	38%	2	24%
		Yes	0		0		0		0	
	Long‐term care	No	5	95%	2	40%	0	0%	1	50%
		Yes	95		24	25%	1	4%	2	8%
Staff		No	56	56%	11	20%	0	0%	0	0%
		Yes	72		5	7%	0	0%	0	0%
Total		No	85	66%	21	25%	3	14%	3	14%
		Yes	167		29	17%	1	3%	2	7%

^a^ Strata size as denominator.

^b^ Respective number of cases as denominator.

Between 11 January 2021 and 26 January 2021, mainly the day‐care was affected with nine detected cases (8/24 (AR = 33%) among care‐receivers and 1/6 (AR = 17%) staff). It was subsequently closed. The outbreak also affected the long‐term care wards and resulted in 26 (AR = 26%) cases among long‐term residents and 15 (AR = 12%) among staff.

**FIGURE 1:**
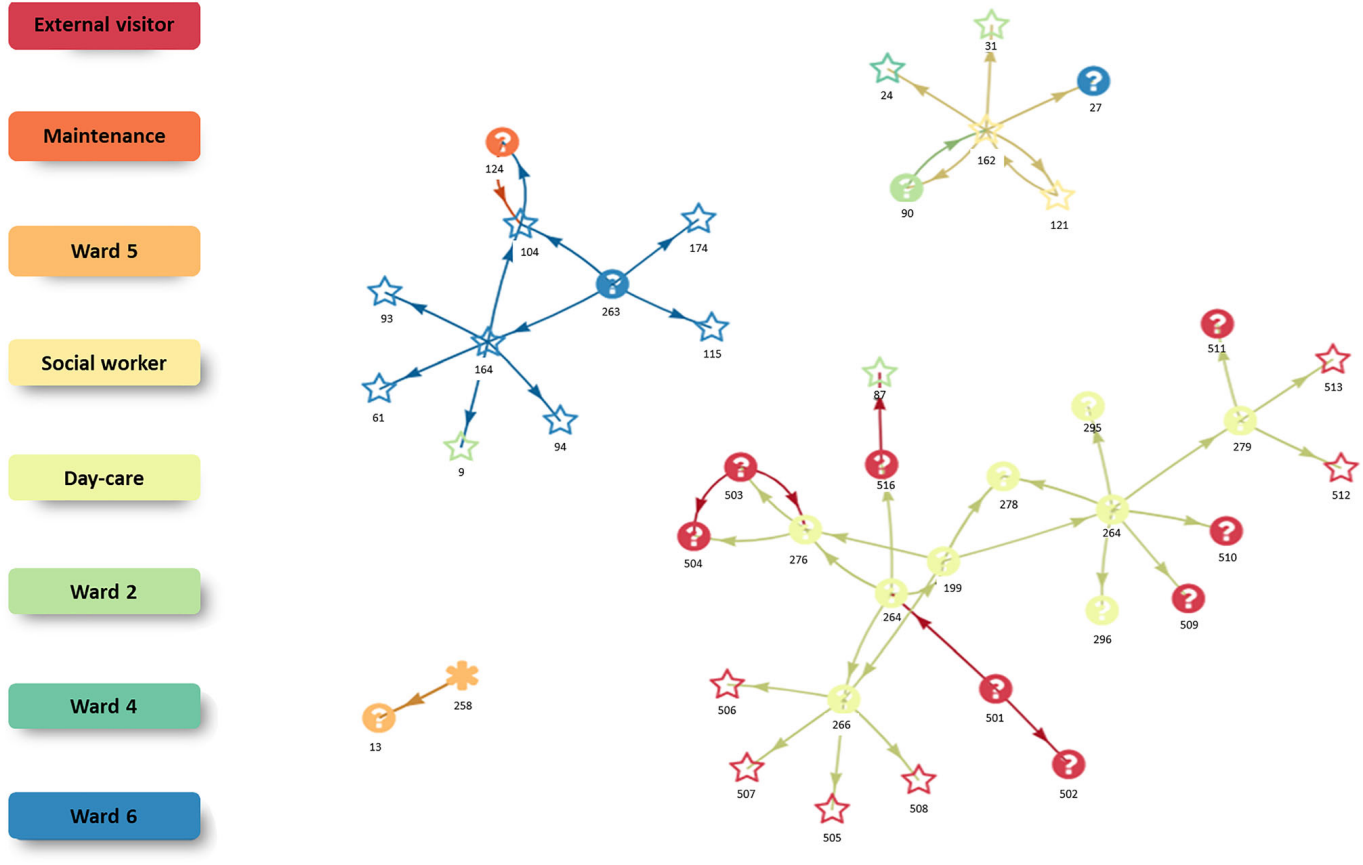
Contact network of PCR‐positive cases of a SARS‐CoV‐2 Alpha outbreak in a nursing home in Osnabrück, Germany, Jan–Mar 2021. Star: Alpha (PCR typing). Asterisk: wild type (PCR typing). Question mark: No PCR typing performed. Number below symbol: ID. Colour represents the area of residence or activity within the nursing home or external for visitors or family members.

The contact network analysis (Figure 1) suggests four different clusters: one cluster with a wild‐type variant and three clusters with an Alpha variant. The largest cluster contains nine cases of day‐care visitors and staff, 14 externals (visitors or household members) and one case of a long‐term resident on ward 2. The latter case was linked to an external visitor, thus connecting a visitor of the day‐care to ward 2. Further, one case among day‐care visitors resulted in up to four cases among household members. Another cluster was mainly limited to ward 6. A third cluster revealed that one case among social care workers was supposedly linked to a total of five cases (one social worker, two cases on ward 2, one case on ward 4 and one case on ward 6). The last cluster consists of two cases of which one case harboured the wild‐type variant and no typing information for the second case.

### 3.3 Reduced SARS‐CoV‐2 infections

All cases with a diagnosis before 25 January 2021 were unvaccinated. Among 29 fully vaccinated cases, the date of diagnosis was 7–11 days after the second vaccine dose in 14 cases (14/29 [48%], all residents) while 15 (52%) were diagnosed 20 or more days post‐vaccination. SARS‐CoV‐2 infections were diagnosed more frequently among unvaccinated than vaccinated residents (Table 1, p = 0.46) and staff (Table 1, p = 0.06). The VE against infection after ≥7 days of two doses of BNT162b2 was 56% (CI: 15–77%, p = 0.04), adjusted for resident/staff status and sex.

### 3.4 Reduced viral load

Ct values at diagnosis were on average 3.04 cycles (p = 0.38) higher among vaccinated than unvaccinated cases. The Ct value at diagnosis increased with time since the second vaccine dose: With an interval of 14 days or higher between second vaccination and sampling, Ct values were on average 3.04 cycles higher (p = 0.12) compared with the shorter interval; with an interval of 21 days or higher, Ct values were on average 4.5 cycles higher (p = 0.08) compared with the shorter interval. Ct values did not differ between males and females (p = 0.60). Typing results were available for 28/50 (56%) samples with 1/28 wild‐type and 27/28 Alpha variants.

### 3.5 Reduced SAR

We analysed 14 households of SARS‐CoV‐2‐positive staff (five vaccinated and nine unvaccinated). No household member was vaccinated in keeping with vaccination prioritization.^4^ Household members with an unvaccinated index case had a higher SAR (67%, 12/18) than household members of vaccinated SARS‐CoV‐2‐positive staff (22%, 2/9, p = 0.046, Table 2).

**TABLE 2:** Secondary SARS‐CoV‐2 cases and secondary attack rate (SAR) in households of SARS‐CoV‐2‐positive staff, stratified by vaccination status, during a SARS‐CoV‐2 Alpha outbreak in a nursing home in Osnabrück, Germany, Jan–Mar 2021

Vaccination status of index staff case	Household members		Secondary attack rate		
	Total (N^a^)	Infected (n^b^)	n/N	SAR	p value
Vaccinated	9	2	2/9	22%	0.046*
Unvaccinated	18	12	12/18	67%	
Total	27	14	14/27	52%	

^a^ N: total household members, excluding household members infected within 6 months prior to the infection of the staff index case and excludes household members who isolated separately from the staff index case.

^b^ n: infected household members.

### 3.6 Vaccination coverage and attack rate among staff

Staff with close physical contact to residents had lower VC (27/59, 46%) and higher AR (17%, CI: 10–28%) than staff working on wards (VC = 29/48, 60%; AR = 8%, CI: 3–20%) and outside of wards (VC = 16/21, 76%; AR = 10%, CI: 3–30%, Table 3). Among the vaccinated staff, those working on wards had the highest AR (AR = 14%, CI: 6–30%) compared with those with close physical contact (AR = 4%, CI: 0.1–18%) or not working on wards (AR = 0%, CI: 0–19%).

**TABLE 3:** Attack rates among staff stratified by their proximity to care‐receivers and vaccination status during a SARS‐CoV‐2 outbreak in a nursing home in Osnabrück, Germany, Jan–Mar 2021

Proximity to care‐receivers	Vaccination status	Total N (%)	SARS‐CoV‐2 infection	Attack rate (95% CI^a^)
With close physical contact	Vaccinated	27 (46%)	1	4% (0.1–18%)
	Unvaccinated	32 (54%)	9	28% (16–45%)
	Total	59 (100%)	10	17% (10–28%)
On wards	Vaccinated	29 (60%)	4	14% (6–30%)
	Unvaccinated	19 (40%)	0	0% (0–17%)
	Total	48 (100%)	4	8% (3–20%)
Outside of wards	Vaccinated	16 (76%)	0	0% (0–19%)
	Unvaccinated	5 (24%)	2	40% (12–77%)
	Total	21 (100%)	2	10% (3–30%)

^a^ 95% confidence interval.

Among the different occupations, specialized nurses (VC = 12/23 = 52%; AR = 4/23 = 17%), nursing assistants (VC = 10/19 = 53%; AR = 4/19 = 21%), nursing interns (VC = 3/9 = 33%; AR = 2/9 = 22%) and kitchen staff (VC = 7/10 = 70%; AR = 2/10 = 20%) had the highest AR, while their VC varied (Table 3).

### 3.7 Outbreak management

Staff had to wear FFP2 masks inside the nursing home at all times. Before the outbreak, all staff conducted daily RADT. Long‐term residents were tested occasionally, for example, when showing COVID‐19‐related symptoms. On 04 February 2021, the LPHA implemented PCR serial testing for all residents and staff every 5 days until 18 March 2021, when two consecutive PCR serial tests had negative results for all tested individuals. Among long‐term residents, non‐cases could move within their ward, but contacts between wards were minimized. Close contacts were quarantined in their rooms. All symptomatic or PCR‐positive long‐term residents were isolated as a cohort on a designated ward. Case isolation ended at the earliest after 14 days and if asymptomatic and PCR‐negative. No visitors were allowed between 03 February 2021 and 18 March 2021. Unvaccinated close contacts among long‐term residents were not vaccinated because of lacking evidence for COVID‐19 vaccines as a post‐exposure prophylaxis. Staff with a positive PCR had to isolate in their household for at least 14 days until they were symptom‐free and the PCR was negative.

### 3.8 Potential source of the outbreak

Typing detected two SARS‐CoV‐2 strains (one wild‐type and 27/28 Alpha variant), suggesting at least two introductory events. The vaccination team tested negative with daily RADT and with weekly PCR. Therefore, we excluded the vaccination team as a source. Potentially, the outbreaks in the day‐care and the permanent care were linked as described above by contact network analyses: An external health‐care worker (ID‐516, Figure 1) visited a presumably highly infectious case (ID‐264, Ct value 11) from the day‐care on 13 January 2021. ID‐516 was RADT‐positive on 16 January 2021, suggesting that the visit to ID‐87, a resident of the permanent‐care on 15 January 2021 (who tested positive later), was within the infectious period. Further, it is possible that staff, for example, the first infected staff on 31 January 2021, introduced SARS‐CoV‐2 infections. However, sequencing data were unavailable to further delineate possible transmission chains. The other 165 visitors of the nursing home had no timely relevant SARS‐CoV‐2 infections and were therefore excluded as potential sources. Although the source remains unclear, we excluded the vaccination team and most visitors as sources.

## 4 DISCUSSION

Day‐care receivers had the highest AR and hospitalization rate. Due to the vaccine prioritization policies at that time,^4^ they were not eligible for COVID‐19 vaccination. Considering that they faced a high vulnerability to infections, hospitalization and death, future vaccination strategies should consider to group day‐care receivers with residents of nursing homes.

In our study, the adjusted VE of two doses of BNT162b2 was moderate against infection and overall lower than in previous studies from similar settings.^7^, ^8^, ^9^ Since this cohort was PCR‐tested every 5–6 days throughout the outbreak, we believe that the risk for missing asymptomatic cases was minimal, while in other studies, under‐ascertainment of asymptomatic infection may have occurred. Furthermore, half of the vaccinated cases were diagnosed within 7–11 days after the second vaccination; thus, infection occurred prior to attaining full immunity. However, we did not observe a higher VE among cases with a longer interval between the second vaccine and diagnosis, in line with findings from the United Kingdom.^10^ Our analysis is limited by the small sample size. Additionally, the individual risk of infection possibly changed over time with the implementation of non‐pharmaceutical control measures, such as separating cases from non‐cases, thereby potentially biasing the VE results of our study.

We found a lower Ct value among vaccinated cases diagnosed ≥21 days after the second vaccine (6 weeks after the first) compared with those with shorter intervals. One study assessing a similar study population^11^ found lower viral loads already 4 weeks after the first vaccination. However, the authors pooled different Ct values from all available PCR‐positive results, which may have impacted the inter‐assay comparability,^12^ especially considering N‐gene dropouts with the Alpha variant.^13^

Our results suggest that while transmission was reduced, close contacts of vaccinated persons with break‐through SARS‐CoV‐2 infections remained at risk for infection, as shown in previous studies for household members of healthcare workers vaccinated with BNT162b2.^14^ As no samples could be sequenced, we are unable to demonstrate that primary and secondary cases had identical viral strains. Still, these results have strong implications for policy makers because they suggest that limiting infections and transmission among vaccinated people requires adhering to non‐pharmaceutical interventions.

Staff with close contact to residents had the highest total AR across all staff groups, when not considering vaccination status. A British study confirmed that nursing staff in hospitals had the highest odds for SARS‐CoV‐2 infection.^15^ An analysis of the German worker's compensation claims for occupational diseases confirmed that nursing staff are most affected by COVID‐19,^16^ especially before vaccination was introduced. We observed that most SARS‐COV‐2 infections occurred among unvaccinated staff. To our knowledge, no study assessed the AR among staff within nursing homes.

The VC of staff with close contact to care‐receivers was the lowest among staff groups despite their intense contact to vulnerable groups. Overall, VC among staff was lower than among residents. A similar trend occurred in nursing homes in the United States.^17^ A low staff VC was associated with more COVID‐19 cases among residents.^18^ Thus, we need to increase acceptance and demand for vaccines, especially among staff with close contact to vulnerable populations to better protect the vulnerable and themselves against infections and severe outcomes.

The high AR among vaccinated staff working on wards indicates that the vaccine alone protects insufficiently against SARS‐CoV‐2 infections in this setting. Thus, adhering to non‐pharmaceutical interventions, such as physical distancing, hand hygiene, and wearing face masks, remains important regardless of VC during outbreaks.

We assessed the use of RADT in a separate publication.^19^ We believe that frequent PCR testing, isolating cases among long‐term residents on a designated ward and quarantining close contacts of cases among long‐term residents in their rooms contributed controlling this outbreak. It is possible that the outbreak on the day‐care and the stationary ward were linked via a visiting health‐care worker. However, strain typing revealed that at least two different strains were present in this outbreak, suggesting at least two introductory events during a time of a high local COVID‐19 incidence in Osnabrück.

The contact network analysis is limited as the data on contacts was incomplete and only available for the first cases. Therefore, it is possible that clusters with the same variant are linked, if connecting cases were not identified. Despite its limitations, the network analysis reveals important information. First, it shows that the outbreak among day‐care visitors resulted in a high number of secondary cases among household members or other contacts. Thus, it is important to strengthen public health measures among contact persons of day‐care visitors of nursing homes to reduce transmission and to identify subsequent cases early. This can include providing sufficient testing opportunities and strengthening measures, such as physical distancing, hand hygiene, and wearing face masks. Second, cases of the Alpha variant had up to five times more secondary cases than cases of the wild type. As this high number of secondary cases could be partly due to incomplete data in the contact network analysis, a higher number of secondary cases is in line with the higher reproduction number of Alpha compared with the wild‐type variant.^20^ Therefore, contact network analysis in outbreaks can serve as a tool to indicate differences in the reproduction number or provide a first assessment of it. Ideally, they require complete contact data and genomic sequencing data to strengthen the evidence. Third, as it was demonstrated by our analysis, social workers are in contact with long‐term residents from different wards, which puts them at high risk for SARS‐CoV‐2 infection and transmission. Thus, it is important that nursing homes create a working environment for social workers which supports them in reducing infection and transmission as much as feasible.

## 5 CONCLUSION

Two doses of BNT162b2 significantly reduced the risk for SARS‐CoV‐2 infections, viral load and secondary transmission, even within 14 days after the second dose. Among the staff groups with physical contact and those not working on wards, vaccinated staff had lower AR than unvaccinated staff. However, this was not observed for the staff group working on wards. Here, reducing infections among social workers is important to minimize the spread between wards. Overall, vaccination rates need to increase among staff. Future vaccination strategies should consider to group day‐care receivers with residents of nursing homes as day‐care visitors face a high vulnerability to infections, hospitalization and death due to or with COVID‐19. Furthermore, day‐care visitors represent a linkage between nursing homes and the community. As a consequence, they can potentially cause secondary cases in both, the nursing homes and the communities. However, the incomplete protection provided by the vaccine against infection and transmission emphasizes that policy makers cannot rely on vaccination only, but need to implement COVID‐19 containment strategies including non‐pharmaceutical measures even for fully vaccinated persons. Regular and frequent PCR serial testing, isolation of cases among long‐term residents on a designated ward and quarantining contacts should supplement outbreak control interventions.

## CONFLICT OF INTEREST

No conflict of interest declared.

## ETHICS STATEMENT

This outbreak investigation was conducted as part of an administrative assistance procedure under the German Infection Protection Act §4 and thus did not require ethical approval.

## AUTHOR CONTRIBUTIONS

**Emily Dorothee Meyer:** Conceptualization; data curation; formal analysis; investigation; methodology; validation; visualization. **Mirco Sandfort:** Conceptualization; data curation; formal analysis; investigation; methodology; validation; visualization. **Jennifer Bender:** Conceptualization; data curation; formal analysis; investigation; methodology; validation; visualization. **Dorothea Matysiak‐Klose:** Conceptualization; data curation; formal analysis; investigation; methodology; supervision. **Achim Dörre:** Formal analysis; methodology. **Gerhard Bojara:** Data curation; investigation; project administration. **Konrad Beyrer:** Data curation; project administration. **Wiebke Hellenbrand:** Conceptualization; formal analysis; investigation; methodology; supervision.

### PEER REVIEW

The peer review history for this article is available at https://publons.com/publon/10.1111/irv.13051.

## Supporting information


**Figure S1:** Directed Acyclic Graph for the association between vaccination status and SARS‐CoV‐2 infection. Vaccination status: two doses of BNT162b2: yes or no; PHSM: public health and social measures (e.g. physical distancing, hand hygiene, face mask, regular room ventilation)

## Data Availability

The data that support the findings of this study are available on request from the corresponding author. The data are not publicly available due to privacy or ethical restrictions.
