# Supplementary figures and images for: BNT162b2 vaccination reduced infections and transmission in a COVID‐19 outbreak in a nursing home in Germany, 2021
Source: Influenza Other Respir Viruses. 2022 Sep 9;17(1):e13051. doi: 10.1111/irv.13051 (PMC9538000; doi:10.1111/irv.13051)

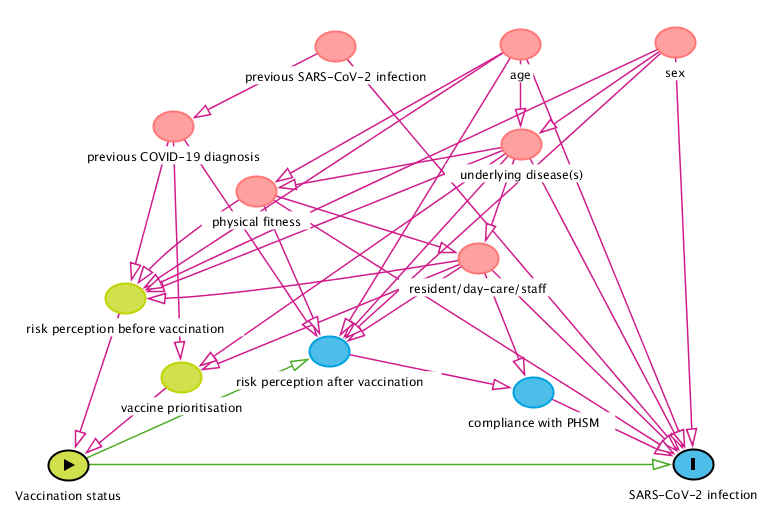

Supplement: Supplementary file 1 — Figure S1: Directed Acyclic Graph for the association between vaccination status and SARS‐CoV‐2 infection. Vaccination status: two doses of BNT162b2: yes or no; PHSM: public health and social measures (e.g. physical distancing, hand hygiene, face mask, regular room ventilation) [file IRV-17-e13051-s001.tif]
